# A Review of Current Literature on Central Retinal Artery Occlusion: Its Pathogenesis, Clinical Management, and Treatment

**DOI:** 10.7759/cureus.55814

**Published:** 2024-03-08

**Authors:** Varun Tiwari, Simerjeet Singh J Bagga, Roshan Prasad, Swapneel Mathurkar

**Affiliations:** 1 Ophthalmology, Jawaharlal Nehru Medical College, Datta Meghe Institute of Higher Education and Research, Wardha, IND

**Keywords:** management, retinal vessels, retinal arteries, retinal artery occlusion, central retinal artery occlusion

## Abstract

The ocular analogue of a cerebral stroke is central retinal artery occlusion (CRAO), a medical emergency concerning the eyes. Most patients experience substantial acute vision loss with a visual acuity of 20/400 or worse, resulting in decreased quality of life (QoL) and decreased functional ability. An impending cerebral stroke and ischemic heart disease are also more likely. The four distinct clinical entities that make up CRAO are non-arteritic CRAO, transitory non-arteritic CRAO, non-arteritic CRAO with cilioretinal artery sparing, and arteritic CRAO. Depending on the CRAO type, clinical traits, visual results, and treatment all vary greatly. Contrary to current belief, there is a spontaneous improvement in the optical field and vision, mainly in the first week. The likelihood of instinctive development in optical acuity in the first seven days varies greatly. The pathogenesis, epidemiology, and medical features of CRAO will be described in this review, along with present and potential management future options.

## Introduction and background

In 1859, von Graefe wrote the first account of central retinal artery occlusion (CRAO). Similar to severe ophthalmic ictus, CRAO too is an ophthalmic emergency. CRAO indicates end-organ ischemia and, typically, the fundamental arterial sclerosis disorders. Due to the same fundamental arterial sclerosis risk factors, there is an increased possibility of cerebral stroke and ischemic heart disease for an individual in the future [1]. Despite being comparable to the severity of brain stroke, there is presently no evidence for therapy that guidelines have approved. The "standard" therapies still used today include methylprednisolone, intravenous (IV) acetazolamide, mannitol, systemic pentoxifylline, sublingual isosorbide dinitrate, hyperbaric oxygen (O_2_) therapy, anterior chamber paracentesis, globe compression, and ocular massage [2].

There is disagreement over the precise location of CRAO. The most frequent reason for CRAO is embolization, and it is mostly caused by carotid artery disease, which is frequently caused by atherosclerotic plaques. Heart disease and carotid stenosis are two other prominent causes of emboli. Overall, it was found that 10.5% of these emboli were made of calcific material, 15.5% of them were made of fibrin, and 74% of them were made of cholesterol [3].

This review aims to delve into the intricate landscape of CRAO, thoroughly examining its etiopathogenesis, its different types, causes of classical and transient CRAO, and systematic factors for its development, clinical presentation, investigation, and management. The primary objectives of this review are to synthesize existing knowledge of CRAO, highlight its clinical presentation, investigation, and management options, and provide researchers and clinicians with a comprehensive resource to understand and navigate the complexities of CRAO. By addressing these objectives, we strive to contribute to the ongoing discourse on CRAO, fostering informed decision-making in clinical practice and guiding future avenues of research.

## Review

Methodology

A primary literature search was carried out using the terms "central retinal artery occlusion," "retinal artery occlusion," "retinal arteries," "retinal vessels," and "management" in the databases MEDLINE, Embase, and PsycINFO. Given that the CRAO is the article's principal emphasis, clinically pertinent systematic reviews, randomised controlled trials, and open studies published in peer-reviewed journals were considered. Articles published between 2010 and 2023 were included in our study to ensure the inclusion of recently published articles, which provide value for comprehensive research. Studies that were not in English, duplicate articles, articles published in non-peer-reviewed journals, articles that had other vascular disorders or had other arteries or branches in focus, and those with missing or inaccessible full texts were excluded. Table 1 shows the summary of the included studies.

**Table 1:** List of included studies in the review. CRAO: central retinal artery occlusion; CRA: central retinal artery; NA-CRAO: non-arteritic CRAO; VA: visual acuity; GCA: giant cell arteritis; IV: intravenous; IA: intra-arterial; tPA: tissue-type plasminogen activator; RCT: randomised controlled trial; IAF: intra-arterial fibrinolysis; O_2_: oxygen

Sr. no.	Author	Year	Type of study	Finding
1	Hayreh [1]	2018	Review	Von Graefe in 1859 wrote first about CRAO. Despite this, an adequate treatment is yet not available.
2	Jayasinghe et al. [2]	2022	Review	Similar to severe ophthalmic ictus, CRAO too is an ophthalmic emergency. Yet, presently, no therapy that guidelines have approved is available.
3	Farris and Waymack [3]	2023	Book	CRAO is the sudden blockage of the CRA, which results in hypoperfusion to the retina and a rapid progressive cell death, leading to loss of vision. An embolus is the commonest reason for this blockage.
4	Dagra et al. [4]	2024	Review	The CRA and its branches supply the inner retina, composed of the layers of ganglion cells, retinal nerve fibres, and inner plexiform cells.
5	Kim et al. [5]	2020	Retrospective study	The cilioretinal artery is present in 20-25% of patients where it still perfuses the macula, even in acute cases of CRAO, thus maintaining a good vision in the CRAO patients with cilioretinal artery.
6	Varma et al. [6]	2013	Review	Although the cilioretinal artery-sparing CRAO is the commonest CRAO, the majority of the eyes only retain a small central vision field, and the prognosis is generally poor due to the variable size of cilioretinal arteries and the region they supply.
7	Tobalem et al. [7]	2018	Review	Duration of CRAO in relation to damage to the retina. Most commonly, retinal infarction happens within 12-15 minutes of complete CRAO.
8	Liu et al. [8]	2023	Review	93.2% of patients presenting with NA-CRAO have VA equivalent to finger counting or worse, and only 22% of patients with NA-CRAO show any improvement within a week of onset.
9	Hayreh and Zimmerman [9]	2005	Cohort study	Visual outcome in NA-CRAO with cilioretinal artery sparing. In more than 65% of patients, VA improves significantly, and 20% of the affected eyes retain 20/40 or greater VA seven days after onset.
10	Hayreh [10]	2021	Review	Arteritic CRAO affects 4.5% of persons, having the poorest prognosis among all four CRAOs. GCA is the most frequent cause of arteritic CRAO.
11	Hayreh [11]	2011	Review	The most common cause of CRAO is embolism. Plaques form on the carotid artery walls due to high cholesterol, especially low-density lipoproteins, a major cause of embolism leading to CRAO.
12	Rudkin et al. [12]	2010	Retrospective study	A previously unidentified vascular risk factor is frequently present in patients with CRAO.
13	Jusufovic et al. [13]	2019	Editorial	The retinal microvasculature, the source and type of emboli, and causes other than emboli, such as intimal dissection, thrombosis in situ, primary antiphospholipid syndrome, sickle cell disease, and deficiency of proteins C and S, are additional factors that explain the lack of effect of reperfusion therapy in CRAO.
14	Hakim and Hakim [14]	2019	Review	Typically, CRAO manifests as an abrupt, painless monocular vision loss. A visual field deficiency is present in 74% of patients with a Snellen VA of counting fingers or worse.
15	Feldman et al. [15]	2023	Review	History, general and physical examination, signs, and clinical diagnosis in CRAO patients.
16	Murphy-Lavoie et al. [16]	2022	Review	The prognosis for recovery of VA is miserable, and this loss of vision is typically severe and irreversible. The severity, duration, and degree of responsiveness to treatment of the patient's symptoms all affect the prognosis after treatment.
17	Mehta et al. [17]	2017	Review	As of now, there isn't clear evidence to say that IV/IA fibrinolytics are helpful for acute CRAO. Large-scale, multicenter RCTs are required to clarify how different acute therapy options fit into the overall management of CRAO.
18	Celebi [18]	2021	Review	Since CRAO occurs so infrequently, compared to other ischemia episodes, it is hard to conduct large-scale RCT, which makes finding a cure difficult. Thus, standard treatments are still being carried out, and hyperbaric O_2_ therapy is being tried to find its effectiveness in CRAO.
19	Takai et al. [19]	2013	Retrospective study	Both monotherapy and combination therapy have been utilized with conservative treatment modalities for acute CRAO. The mean rate of improvement in VA was 15-21%.
20	Hayreh [20]	2014		To dissolve fibrino-platelet obstruction of the CRA in NA-CRAO is the aim of thrombolysis in CRAO. Since 1984, arteries in CRAO have been re-canalized via local IAF. Small retrospective investigations have shown its effectiveness.
21	Mac Grory et al. [21]	2020	Cohort study	This study shows that in patients with acute CRAO, there is a chance of a good visual outcome when IV alteplase is administered within four and a half hours of the onset of symptoms.
22	Dumitrascu et al. [22]	2020	Systematic review	There have been reports of IV thrombolysis with alteplase; 54% of these patients received IV tPA within four and a half hours of the onset of symptoms, and none of them experienced ocular or cerebral haemorrhage.
23	Mac Grory et al. [23]	2020	Review	Current evidence shows that acute CRAO should be handled as an emergency when it comes to treatment. Additional RCT should be conducted to investigate the various drugs now available for IV thrombolysis within the four-and-a-half-hour timeframe to treat this debilitating illness.
24	Schumacher et al. [24]	2010	RCT	In comparison between IAF and tPA therapies, similar results are seen, but a higher percent of adverse reactions are associated with IAF; we cannot recommend IAF for treating acute CRAO, while tPA shows promising results.
25	Sim and Ting [25]	2017	Review	Many treatment options are said to help with acute CRAO, but there isn't enough proof to back them up. Even though CRAO has a poor prognosis, attempts should be made to restore vision, regardless of the therapy utilized, as soon as symptoms appear, ideally within four hours.
26	Jung et al. [26]	2016	Cohort study	The approach for subacute management is to avoid secondary neovascular complications of the eye. The potential for neovascularization and eventual glaucoma is another complication of CRAO. There is no agreement on the optimum post-CRAO follow-up strategy for identifying ocular neovascular problems and effectively managing CRAO.
27	Mac Grory et al. [27]	2021	Review	The approach for long-term management is to avoid further episodes of vascular ischemia that might harm the eye or cause another end-organ damage. The proposed vascular examination and investigations must be carried out since two patients in this study developed systemic ischemia events.
28	Cugati et al. [28]	2013	Review	CRAO is an ocular emergency. To avoid further medical comorbidities, CRAO patients must be evaluated to have the same atherosclerotic risk factors that predispose them to peripheral, cardiovascular, and cerebrovascular illnesses. These risk factors need to be actively assessed. The effectiveness of the current acute management for acute CRAO in improving eyesight is limited, and research indicates that treatment for CRAO needs to be started as soon as possible, possibly within six hours of the onset of symptoms.
29	Murphy-Lavoie et al. [29]	2012	Review	Every patient who presents within 24 hours of diminished vision and has indicators pointing to CRAO should be given supplementary O_2_. They assert that early treatment may prevent retinal deterioration.
30	Madike et al. [30]	2022	Review	When a patient complains of sudden vision loss in one eye and is thought to have CRAO, a thorough history should be obtained. The duration of the patient's symptoms, any systemic symptoms that might point to GCA, and any related neurological symptoms should all be included in this history. Eliminating tPA contraindications, such as recent surgical procedures or significant bleeding, is also crucial. The next step should be a thorough examination of the eyes, ideally by an ophthalmologist. In consultation with the stroke team, IV tPA should be considered if the patient has experienced vision loss for less than four and a half hours.

Anatomy and etiopathogenesis

The central retinal artery (CRA) supplies the optic disc's surface layer. Here, CRA gets branched into inferior and superior, which again branches into temporal and nasal and provides blood to the inferior, superior, nasal, and temporal quadrants of the retina. The outer retina is supplied by the choriocapillaris of the choroid, which divides from the ciliary artery. Given that both the CRA and ciliary artery arise from the ophthalmic artery, thus, both CRA and ciliary artery should be functioning to maintain retinal function [4].

The presence of a cilioretinal artery creates a significant variation among CRAO patients and creates a difference in the visual acuity (VA) of CRAO with and without a cilioretinal artery. It is present in 20-25% of patients where it still perfuses the macula, even in acute cases of CRAO, thus maintaining a good vision in CRAO patients with cilioretinal arteries. This does not always happen, though [5]. In an examination, 35 of the 260 CRAO-affected eyes were found to have cilioretinal arteries. Around 60% of them had an initial VA of 6/30 or below. These subpar findings are due to the variable size of cilioretinal arteries and the region they supply. Therefore, in CRAO patients without a cilioretinal artery, the inner retinal layers' thickness will decrease due to CRAO infarcts cutting off the retina's blood supply. However, owing to the presence of a cilioretinal artery, it can maintain the thickness, depending on the proportion of retina it supplies in a CRAO patient with a cilioretinal artery [6].

The exact location where CRAO occurs is debated. But, anatomical studies show that CRAO is usually because of CRA everlasting obstruction caused by an affected embolus as it penetrates the optic nerve sheath, the CRA's narrowest point (not at the lamina cribrosa, as is often incorrectly defined). Rarely, vasculitis, persistent systemic inflammatory diseases, and thrombophilia cause CRAO. The plaques of the heart or the carotid artery serve as the emboli's primary point of origin. Overall, the composition of these emboli is found to be 74% cholesterol, 10.5% calcific debris, and 15.5% fibrin. An occlusive thrombus at the level directly posterior to the lamina cribrosa is also likely to be the aetiology of CRAO [1,6].

After the CRA is blocked, the retina's ability to heal is determined by the retinal ischemia tolerance time, which is more significant than whether the offending thrombus or embolus is removed. Studies on electrophysiology, histology, and morphometry concluded that CRAO of 97 minutes had no appreciable adverse effects on old, atherosclerotic, hypertensive rhesus monkeys. However, between 105 and 240 minutes, visual evoked potential revealed different degrees of irreversible partial retinal tear. Significant inter-individual variation was found amid the extent of the CRAO and the severity of retina injury. At 240 minutes, there was significant, irreversible retinal damage in every eye, as well as complete or nearly complete optic nerve atrophy and nerve fibre loss. Therefore, the window of opportunity for intervention is suggested to be constrained and inversely correlated with the degree of recovery. It's unclear how long a retina can withstand damage before irreparable damage sets in, although it appears to be no more than four hours [5-7].

Classification of CRAO

CRAO has long been regarded as a single illness. Nevertheless, four unique clinical entities make up CRAO.

Permanent Non-arteritic CRAO (NA-CRAO)

It is the most prevalent CRAO, found in over two-thirds of CRAO patients. More than two-thirds of cases of CRAOs are brought on by platelet-fibrin thrombi and emboli, which are the consequence of atherosclerotic disease. Atherosclerosis, diabetes mellitus, coronary artery disease, carotid artery disease, transient ischemic attacks (TIAs) or cerebral vascular accidents, and tobacco use are risk factors for NA-CRAO. Compared to the general population, patients with CRAO have been reported to have a much higher prevalence of these [6]. While 93.2% of patients presenting with NA-CRAO have VA equivalent to counting fingers or worse, only 22% of patients with NA-CRAO show any improvement within a week of onset. A cherry-red patch, greyish retinal oedema, retinal artery constriction, and little to no residual retinal circulation on fundus fluorescein angiography (FFA) are typical symptoms [8].

Transient NA-CRAO

In animal models, temporary CRAO has also been linked to transient vasospasm brought on by platelets' release of serotonin on atherosclerotic plaques. It is analogous to an ophthalmic TIA, has the best prognosis for VA, and accounts for 15-17% of CRAOs. In this instance, the duration of CRAO may range from a few minutes to many hours, subject to the source of obstruction. The symptoms go away when the blood supply to the CRA is restored. Around 82% of affected individuals eventually show improvement in their VA, and 37.9% reach a VA of 20/40 or better within seven days after the onset of symptoms. Emboli migration, vasospasm, and a sudden drop in intraocular pressure (IOP) or increase in perfusion pressure are a few examples of the causative variables. The degree to which the VA will be lost will differ significantly from any other type of CRAO, depending on how long the transient CRAO lasts. Yet, cerebrovascular accidents and ocular recurrence are possible within five years of receiving treatment for transient NA-CRAO, while ipsilateral stroke occurs in 22.2% of patients [6,8].

NA-CRAO With Cilioretinal Artery Sparing

Around 14.3% of CRAO patients have this condition. A cilioretinal artery is present in 20-25% of patients where it still perfuses the macula, even in acute cases of CRAO. It substantially impacts the flow from the retina and thus helps in a good prognosis for VA in patients of CRAO with and without a cilioretinal artery. This helps maintain good vision in CRAO patients with cilioretinal arteries. In 67% of affected eyes, VA improves significantly, and 20% of all eyes retain 20/40 or greater VA seven days after onset [6-9].

Arteritic CRAO

The prognosis is worse in people with arteritic CRAO, which affects 4.5% of patients. The most frequent cause is giant cell arteritis (GCA), an idiopathic vasculitis that mostly affects the upper body and head in older persons and almost invariably results in small- to medium-sized artery stenosis and occlusion. To diagnose GCA, a superficial temporal artery biopsy is crucial. Constricted intimal hyperplasia, rupture of the internal elastic lamina, and infiltration of multinucleated giant cells are typical signs; it is important to note that the absence of large cells on biopsy does not rule out GCA. The majority of eyes that have CRAO caused by GCA also have arteritic anterior ischemic optic neuropathy. A raised C-reactive protein level, an elevated erythrocyte sedimentation rate, fever, systemic headache, otalgia, and chewing pain are indicators that support the diagnosis. Both the CRA and cilioretinal arteries are obstructed due to the ophthalmic artery's role in the pathophysiology of arteritic CRAO, which prevents improvements in VA unless high-dose corticosteroids are given [7,10].

Systemic factors contributing to the development of CRAO

Plaques form on the carotid artery walls due to high cholesterol, especially low-density lipoproteins, or "bad cholesterol," a major cause of embolism and CRAO. Two approaches can cause CRAO in people with carotid artery disease. First, embolization is the most frequently observed cause of CRAO. Second, a considerable stricture (70% or added) or whole blockage of the internal carotid artery drastically lowers the blood circulation to the eyes, leading to the growth of CRAO. Around 18% of CRAO patients in one research had internal carotid artery stenosis of higher than 80%. Numerous studies have shown a connection between CRAO and arterial hypertension and diabetes. CRAO can result from a variety of cardiac diseases due to heart-related embolism. Additionally, CRAO has been seen to occur after invasive cardiovascular procedures. It has been reported that CRAO is related to GCA, vasculitis, and chronic systemic autoimmune diseases. Several controversial papers assert that thrombophilia has a role in the growth of CRAO. Seldom, haematological disorders such as orbital lymphoma, leukaemia, systemic non-Hodgkin's lymphoma, and sickle cell hemoglobinopathies have been related to CRAO. Additionally, postoperative reports of CRAO have been made succeeding a variability of operating events, including those requiring subsequent eye, orbit or head injuries, face and retrobulbar injections, peribulbar anaesthesia, and intraocular gas injections used as tamponades for rhegmatogenous retinal detachment. After hemodialysis, central artery occlusion is a recognized procedure. It has been recognized that receiving hemodialysis frequently causes blood pressure to drop dramatically. These people almost invariably have significant vasculopathy as well. Incontinentia pigmenti, Fabry's disease, snake bite, cocaine abuse, use of oral contraceptives, nephrotic syndrome, migraine, and other uncommon, unrelated disorders are only a few of the uncommon, unrelated conditions linked to CRAO [11-13].

Clinical presentation

**Figure 1:**
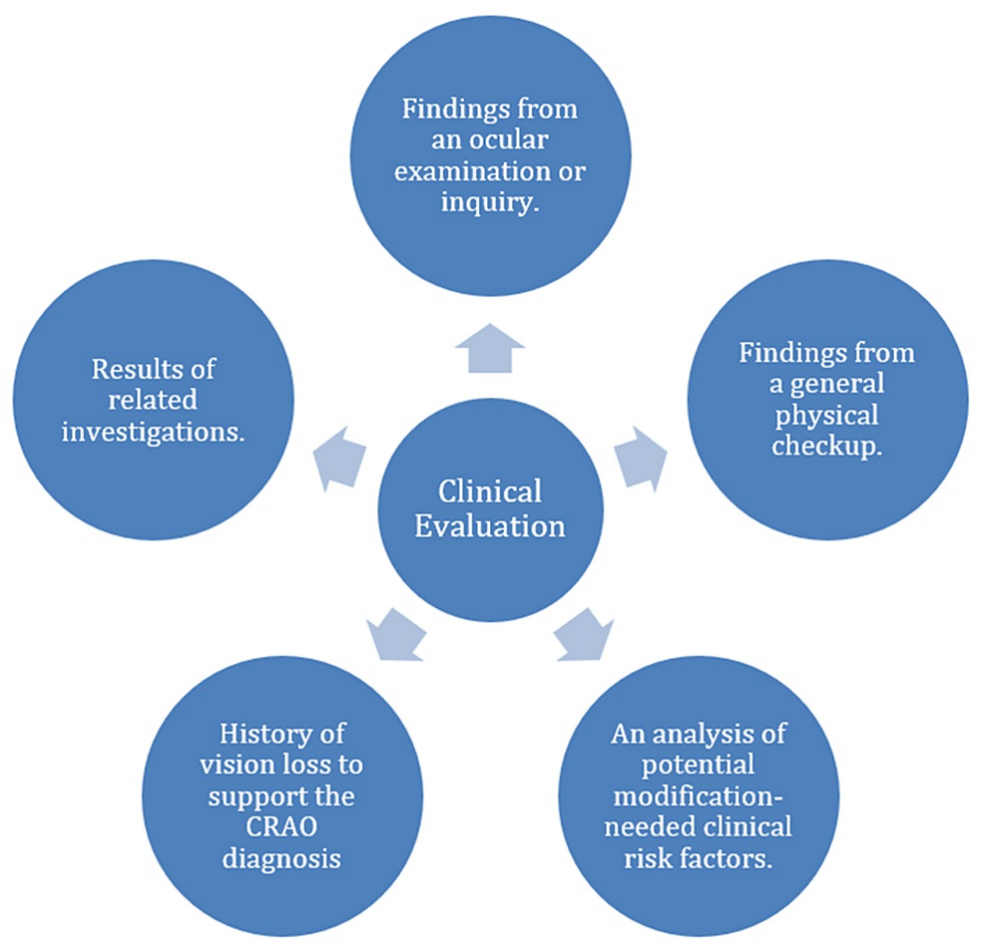
Aspects to be included while assessing CRAO clinically. This figure is self-made by the author. CRAO: central retinal artery occlusion

Figure 1 below shows aspects to be included while assessing CRAO clinically.

History

Typically, CRAO manifests as an abrupt, painless loss of monocular vision. A visual field deficiency is present in 74% of patients with a Snellen VA of counting fingers or worse. Central vision preservation may be possible if a cilioretinal artery is present. Thus, it is recommended to assess atherosclerotic risk factors, which include diabetes, hyperlipidemia, a family history of cerebrovascular and cardiovascular disease, atherosclerotic cardiac or cerebrovascular disease, valvular heart disease, and transient ischemic events, such as transient monocular blindness, TIAs, or anginal symptoms. When no atherosclerotic risk factors exist, additional less prevalent causes should be investigated, particularly in young patients. These include the usage of ocular cicatricial pemphigoid/intravenous (OCP/IV) medications, myeloproliferative illnesses, vasculitis, sickle cell disease, and hypercoagulable conditions [14].

General Examination

This can be split into two parts: first, an evaluation of vascular risk, and second, the ocular findings to corroborate the differentials and rule out additional potential sources.

Evaluation of cardiovascular risk: To identify potential causes, a physical examination should be performed with special attention to the cardiovascular system. The radial pulse rate and rhythm are significant because atrial fibrillation, which increases the risk of embolic events, is suggested by rhythmically irregular heartbeats. Because CRAO and hypertension are related, taking blood pressure is crucial. To rule out the possibility of temporal arteritis, scalp pain and a nodular temporal artery examination should be carried out. A complete physical examination should be performed on younger individuals to rule out any autoimmune connective tissue illnesses that could put them at risk for developing vasculitis [6,15].

Ocular evaluation: Fundoscopic results in CRAO differ according to the kind and the duration after the occurrence. Early research conducted within seven days after CRAO revealed the following findings: 90% of eyes showed cherry-red patch, 19% cattle truck appearance, 32% retinal artery attenuation, 58% posterior pole retinal opacity, 22% optic disc oedema, and 39% pallor. Later, FFA examinations showed 91% of eyes with optic atrophy, 58% with attenuation of the retinal arteries, 18% with cilioretinal collaterals, and 11% with changes to the macular retinal pigment epithelium. The FFA findings of transitory CRAO were very varied. According to the degree of VA loss and fundoscopy, CRAO may also be categorized into phases: total, subtotal, and incomplete CRAO. This aids in prognosis prediction. Another study concluded that on fundoscopic inspection, 20% of patients had intra-arterial (IA) emboli. This is crucial because establishing the aetiology of CRAO might be aided by the morphological appearance of emboli. Small, yellow, refractile plaques, also known as "Hollenhorst plaques," for example, are indicators of cholesterol emboli, while small, pale bodies are indicators of fibrin-platelet emboli, and single, white, non-scintillating plaques are indicators of calcific emboli in the proximal retinal vasculature [6,15].

Contralateral eyes should always be examined to identify underlying diseases, such as hypertensive retinopathy, alterations in the arterioles, or prior vaso-occlusive disorders. Other ophthalmologic investigations may point to the CRAO's cause. The presence of minor vascular disease may be suggested by ophthalmoscopy results of sickle cell disease or hypertensive retinopathy. A physical exam of the cardiovascular system should be the major focus to evaluate vascular risk and identify likely causes. Since irregular beats indicate atrial fibrillation, which raises the risk of embolic events, the frequency and regularity of the radial pulsation are significant. Measuring blood pressure is important since CRAO and hypertension are related. Young people should get a thorough assessment to identify autoimmune connective tissue diseases that might be risk factors for vasculitis [6,15].

Management

The phrase "a sickness without a cure has many cures" perfectly describes CRAO. Before considering CRAO treatments, it should be noted that the disease's natural course indicates that CRAO patients may experience spontaneous visual improvement. However, the kind of CRAO and its duration greatly affect the degree of improvement. Only 10% of individuals with spontaneous reperfusion get an increase of three lines on a Snellen chart or another significant improvement. CRAO patients often have VAs of 20/200 or below [16].

Acute Management

The approach for acute management is that CRA's ocular perfusion should be attempted to be restored. However, people are seldom treated for CRAO with acute care, and there is no accepted course of treatment or model of care. These are the main obstacles to successful treatment for CRAO. There are now two major therapy options for the treatment of acute NA-CRAO. The first are conventional non-invasive procedures, and the second method is administering thrombolytics intravenously or intra-arterially [17].

Standard non-invasive treatments: People may utilize systemic pentoxifylline, sublingual isosorbide dinitrate, or a carbogen inhalation, such as hyperbaric O_2_, to raise blood O_2_ levels and enlarge retinal arteries. To attempt to remove any emboli, rub the eyes. The tension inside the eye is decreased to raise the pressure in the retinal arteries, which in turn elevates the perfusion pressure using IV mannitol, acetazolamide, anterior chamber paracentesis, as well as the expulsion of aqueous from the eye in small amounts. Globe compression, ocular massage, IV acetazolamide, IV mannitol, sublingual isosorbide dinitrate, methylprednisolone, retrobulbar tolazoline, streptokinase, and other anticoagulants are examples of multimodal stepwise conservative strategies [18].

Both monotherapy and combination therapy have been utilized with conservative treatment modalities for acute CRAO. The success rate of this type of therapy ranges from 6% to 49%, with a mean rate of improvement in VA of 15-21%. However, these interventions generally do not alter the outcome more than the disease's natural course. This is because much of the data is observational; thus, some may show an improved result over natural history. Two randomised controlled studies have been conducted to look at conservative therapies for CRAO. They propose that greater external counterpulsation and oral pentoxifylline may be useful in the management of CRAO. Despite evidence of increased retinal perfusion following intervention, this did not increase in VA [19].

To dissolve fibrino-platelet obstruction of the CRA in NA-CRAO is the aim of thrombolysis in CRAO. Similar treatments exist for acute ischemic stroke and coronary artery blockage. Since 1984, arteries in CRAO have been re-canalized via local IA fibrinolysis. Small retrospective investigations have shown its effectiveness. IA fibrinolysis has been demonstrated to be successful in CRAO in a number of open-label observational studies, with up to 60-70% of treated patients reporting an improved VA. Retrospective case-control research unequivocally established that IA thrombolysis within four hours of a stroke generated better visual outcomes than later treatment. When tissue-type plasminogen activator (tPA) was administered intravenously in aliquots for up to 15 hours to CRAO patients between 1999 and 2006, the Johns Hopkins Hospital saw a statistically significant improvement in vision of at least three lines compared to control participants who did not receive thrombolysis. Around 84 people with CRAO participated in the multicenter prospective randomised controlled research known as the European Assessment Group for Lysis in the Eye (EAGLE) within 20 hours of the onset of symptoms. Among the lysis and standard therapy groups, no clinical improvement was substantially different (60% vs. 57.1%). However, the local IA fibrinolysis group had a substantially higher incidence of adverse outcomes than that of the group receiving standard care (37% compared with 4.3%) [20,21].

As per the usual ischemic stroke thrombolysis procedure, IV administration of thrombolysis is also an option. Theoretically, IV administration provides advantages over oral administration, including simpler access and a lower risk of haemorrhagic complications without requiring a specialist interventional radiology setup. The higher risk of strokes and direct vascular damage, the need for a neuro-interventionalist, and the lengthier procedure duration are some of its drawbacks. According to an interventional case study, patients who received concurrent IV heparin and low-dose IA tPA (50 mg) in six and a half hours to help prevent re-occlusion had significant vision improvement of at least three Snellen lines [22].

In studies, acute CRAO patients' eyesight did not noticeably improve after IV tPA at 24 hours. Subgroup analysis, however, revealed that the only patients who got IV tPA within six hours of beginning did so with improvements of greater than three lines. According to this study, up to six hours after CRAO may be the maximal retinal tolerance time for successful reperfusion treatment. But this treatment ought to be given as soon as feasible. The outcomes found by Hattenbach et al. are comparable to those of this six-hour time span. These findings differ significantly from a study's ground-breaking research on rhesus monkeys. Their research shows that the retina suffers irreparable damage 240 minutes after CRAO. Time is of the essence if one considers the data from the aforementioned human and animal research, in addition to prior information on reperfusion in the cardiac, cerebral, and peripheral vascular circulations. Because only a tiny portion of persons maintain viable tissue after 24 hours, the EAGLE study's inability to demonstrate effectiveness can be linked to using that time range [23,24]. Table 2 below summarises all available treatment options and their mechanism of action for managing acute CRAO [25].

**Table 2:** The available treatment options and their mechanism of action for managing acute CRAO. The author re-created the table using information from [25]. IV: intravenous; O_2_: oxygen; IA: intra-arterial; tPA: tissue-type plasminogen activator; Nd:YAG: neodymium-doped yttrium aluminum garnet; IOP: intraocular pressure; CRAO: central retinal artery occlusion; SpO_2_: oxygen saturation

Treatment	Mechanism of action
Pharmacological	
IV mannitol	Reduces IOP
IV acetazolamide	Reduces IOP
Topical anti-glaucoma medications	Reduces IOP
IV methylprednisolone	Reduces retinal oedema (only used for cases of arteritic CRAO)
Sublingual isosorbide dinitrate	Vasodilation; raises blood SpO_2_
Inhalation of carbogen	Vasodilation; raises blood SpO_2_
Pentoxifylline	Vasodilation; raises blood SpO_2_
Hyperbaric O_2_ therapy	Increases blood O_2_ tension
IV or IA recombinant tPA	Thrombolytic
Surgery/procedures	
Globe massage	IOP has fluctuated to dislodge the clot mechanically
Anterior chamber paracentesis	Reduces IOP
Pars plana vitrectomy	Surgical removal of the clot
Nd:YAG laser embolectomy	Lyses or dislodges clot

*Complications* *With* *IV/IA* *Thrombolysis*

Haemorrhage is the main side effect of thrombolysis. In contrast to no incidents in the control group, 8% of the thrombolysis therapy group experienced symptomatic cerebral haemorrhage within 36 hours of treatment, with 4% of those cases being fatal, according to the National Institute of Neurological Disorders and Stroke (NINDS) trial, which included 291 patients. Furthermore, 3% of the treatment group's patients experienced asymptomatic cerebral bleeding within 36 hours of starting therapy, and an additional 3% of the group experienced symptomatic intracranial haemorrhage during long-term follow-up. As such, patients with underlying illnesses that could be fatal in the event of a bleed should not receive thrombolysis. These include the use of oral anticoagulants, liver cirrhosis, stomach ulcers, haemorrhagic diathesis, myocardial infarction, cardiac aneurysm, and uncontrollably high blood pressure [13].

Subacute Management

The approach for subacute management is to avoid secondary neovascular complications of the eye. The potential for neovascularization and eventual glaucoma is another complication of CRAO. In the literature, disagreement exists on the occurrence and genesis of the condition that follows CRAO. In their cohort of 232 individuals with CRAO, a study failed to find a cause-and-effect association between CRAO and ocular neovascularization. The patients who underwent neovascularization lacked any additional clinical traits, such as diabetes or a connection to hemodynamically significant carotid artery stenosis, that might have contributed to the neovascularization in addition to CRAO, according to a study that presented a temporal association between CRAO and neovascularization events. As a result, there is no agreement on the optimum post-CRAO follow-up strategy for identifying ocular neovascular problems and effectively managing CRAO. Normal neovascularization starts eight weeks (maybe 2-16 weeks) following CRAO. Therefore, it is prudent to conduct routine examinations on every affected role with acute CRAO, beginning as soon as two weeks after the occurrence and continuing every month for as long as four months [26].

Long-Term Management

The approach for long-term management is to avoid further episodes of vascular ischemia that might harm the eye or cause another end-organ damage. To stop further cases of ischemia, CRAO treatment must target systemic atherosclerotic risk factors. The proposed vascular examination and investigations must be carried out since two patients in this study developed systemic ischemia events (cerebral stroke and acute coronary syndrome). Therefore, vascular risk factors that can be treated with medication or surgery are commonly undetected in those who report using CRAO. Treatment of risk factors is crucial since this population has a significant risk of repeated ischemia episodes [6,27].

Discussion

A cerebral vascular injury involving the retina is similar to CRAO. As a result, CRAO diagnosis and treatment are relatively comparable to those for TIAs or stroke. The primary blood workup should comprise point-of-care glucose, full blood counts with differential, and coagulation tests (prothrombin time/international normalized ratio (PT/INR), partial thromboplastin time (PTT)). The patient's erythrocyte sedimentation rate and C-reactive protein should be checked to exclude the chances of temporal GCA. High-dosage steroids should be begun immediately if inflammatory markers are elevated and history and physical examinations suggest GCA [28].

A CT of the head without contrast should be performed to exclude cerebral haemorrhage and evaluate whether the patient qualifies for thrombolytic therapy if it has been less than six hours shortly after the onset of symptoms. Based on unique risk factors and histories, additional workup should be considered. Outside of a hospital context, this may entail testing for hypercoagulability, carotid artery duplex, ECG, echocardiography, lipid profile, Rh factor, fluorescent treponemal antibody absorption test, hemoglobin A1c, and Holter monitoring. Consider electroretinography and/or IV fluorescein angiography to confirm the diagnosis. There is disagreement over the best course of action for treating CRAO, while early IV thrombolytic therapy appears promising [3,28].

According to a 2012 literature review, every patient who presents within 24 hours of diminished vision and has indicators pointing to CRAO should be given supplementary O_2_. They assert that O_2_ therapy poses no risks and that early treatment may prevent retinal deterioration. To begin inhaling O_2_, the highest acceptable amount of inspired O_2_ should be utilized (fraction of inspired oxygen (FiO_2_)). Hyperbaric O_2_ therapy should be considered if it is offered [29].

Suggested Management

A complete history should be taken when a patient presents with sudden loss of vision in one eye and is suspected of having CRAO. This history should include the duration of the patient's symptoms, systemic symptoms that may indicate GCA, and associated neurological symptoms. It's also critical to rule out tPA contraindications, such as recent surgeries or severe bleeding. The next step should be a thorough examination of the eyes, ideally by an ophthalmologist. In consultation with the stroke team, IV tPA should be considered if the patient has experienced vision loss for less than four and a half hours. The patient should be started on antiplatelet treatment and be evaluated by the acute stroke unit for workup and secondary prevention if they have experienced symptoms for more than four and a half hours [30].

Limitations and future prospects

The fundamental processes and therapeutic techniques of cerebral ischemic stroke and CRAO are similar. There isn't yet a commonly recognized treatment for CRAO, and different clinicians handle it differently. The evidence on IV tPA for CRAO is still limited due to several factors, such as a lengthy treatment window and ambiguous or imprecise visual recovery outcomes. After discussing the advantages and disadvantages with the patient or surrogate, IV tPA may be a viable treatment for people with CRAO. Regarding improvement in VA, historical techniques like anterior chamber paracentesis, ocular massage, and hemodilution are not helpful. New therapies, like IA tPA and hyperbaric O_2_ at early stages, have potential but need more research [27].

Similar to cerebral ischemic stroke, we must create care systems for the prompt identification, assessment, and treatment of CRAO. Expert evaluation and the start of treatment at outlying centers without access to internal specialists will be made possible by telemedicine. To clearly define the current epidemiology of CRAO, population-based studies are required, as well as additional research to assess the quality of life (QoL) following CRAO in the long run. To potentially enable the use of tPA at delayed time points in certain patients, future research should focus on developing novel indicators of retinal tissue viability that can be deployed in real time and complement current time-based decision-making algorithms. Further research is needed to evaluate novel thrombolytic drugs such as tenecteplase, hyperbaric O_2_ therapy, and novel neuroprotectants that can be used with re-canalization therapy [27].

## Conclusions

The ocular equivalent of a brain stroke is CRAO, which should be treated as an emergency involving the eyes. Cerebrovascular events and CRAO share many of the same risk factors and causes. To reduce the risk of subsequent ischemic episodes and prevent the risk of additional comorbidities, it is important to actively discover the same atherosclerotic risk factors present in CRAO and incline to cardiovascular, peripheral, and cerebrovascular illness. Patients with CRAO must be evaluated for stroke as soon as possible. Even though CRAO has a dismal prognosis, attempts should be made to restore vision as soon as symptoms appear, ideally within four hours of diagnosis, regardless of the therapy used. Similar to cerebral ischemic stroke, we must create care systems for the prompt identification, assessment, and treatment of CRAO. Acute CRA reperfusion, avoiding ocular sequelae, and vascular evaluation to stop future end-organ ischemia are the main goals of effective CRAO therapy.
